# Theoretically informed gender analysis for gambling harm reduction: a New Zealand study

**DOI:** 10.1186/s12954-021-00558-5

**Published:** 2021-11-08

**Authors:** Katie Palmer du Preez, Anna-Marie Paavonen, Maria E. Bellringer

**Affiliations:** 1 Faculty of Health and Environmental Sciences, AUT University, Auckland, New Zealand; 2 Kela, Helsinki, Southern Finland Finland

**Keywords:** Gambling, Gender analysis, Women, Social determinants

## Abstract

**Background:**

Gambling harm affects men and women relatively equally, and gender influences the social determinants of gambling harm. Responses to preventing and minimising women’s gambling harm have been shaped and constrained by population research identifying male gender as a key risk factor for gambling problems. Gender analysis in gambling studies is rare and has lacked theoretical underpinning and coherence, limiting possibilities for gender-responsive and gender-aware harm prevention and reduction activities.

**Methods:**

Two influential qualitative studies of gambling harm in New Zealand (involving total *n* = 165 people who gambled, affected others, community leaders, gambling and community support service providers, policy makers and academics) neglected to explore the role of gender. This study revisited data collected in these studies, using thematic analysis informed by feminist social constructionist theory. The overarching research questions were: How do gender-related issues, notions and practices influence women’s gambling related harm? What are the implications for women’s gambling harm reduction?

**Results:**

Women’s socio-cultural positioning as primary caregivers for families and children constrained their ability to access a range of recreational and support options and increased the attractiveness of local gambling opportunities as accessible and ‘safe’ outlets for stress reduction. Patriarchal practices of power and control within family contexts operated to maintain gambling behaviour, shut down alternative recreational opportunities, and limit women’s autonomy. Consideration of these themes in relation to current health promotion practice in New Zealand revealed that national programmes and strategies appear to be operating without cognisance of these gender dynamics and therefore have the potential to exacerbate or cause some women harm.

**Conclusions:**

This study demonstrates the value of theoretically informed gender analysis for gambling harm reduction research, policy and practice. International guidelines for gender-aware and gender-responsive health research and practice should be engaged as a foundation for strategic and effective gambling harm reduction programmes, projects, research and policy, and as an essential part of developing and implementing interventions for gambling harm.

## Background

Gambling research and intervention practice has tended to focus on young men, who are more likely to develop problem gambling than other demographic groups [1–3]. The health and social costs of gambling are estimated to be substantial when calculated using burden of harm methodologies which elucidate and measure harms occurring for and around people at all gambling severity levels (low-risk, moderate-risk and problem gambling) [4–6]. Gambling-related harm includes but is not limited to: financial hardship, poorer health, psychological and emotional distress, and impaired social and cultural relationships [7]. These issues can linger long after the gambling has stopped, as encapsulated by the notion of ‘legacy gambling harm’ [7]. Gambling-related harm is understood not only in terms of the effects on the person who gambles, but impacts that can occur to family, friends, whānau (extended family), and the broader community [5, 8]. For example, two recent systematic reviews of population, clinical and community-based research have illuminated the multifaceted and complex nature of gambling harms experienced by the significant others of people experiencing gambling problems [9, 10]. Physical and mental health problems are linked to living in a state of uncertainty and fear, social disconnection and material deprivation.

The conceptualisation and study of gambling-related harm has drawn attention to harm experienced by women. In Australia and New Zealand, where large scale studies of the burden of gambling harm have been carried out, the bulk of population-level harm was seen to be occurring around lower gambling risk categories, and the gambling of men and women equally [5, 6]. In the Australian gambling harms study, Victorian women in the low-risk problem gambling severity (PGSI) category were associated with nearly one-third (28.9%) of years of life lost to disability associated with gambling. Women 55 years and over with low-risk gambling behaviour were associated with the largest proportion of harms of any single demographic category (14.5%) [6]. Similar numbers of women and men report experiencing harm from others’ gambling in population studies (e.g. [11]), though women seem more likely to identify their spouse/partner as having a gambling problem than men, e.g. 2.9% cf. 1.5% in the New Zealand population [12]. This is important given the strong association between intimate partner relationship quality and health and wellbeing in Western societies [13]. From a public health perspective, women are at least as deserving of attention as men for gambling harm reduction practices. Adams et al. [14] outlined opportunities to respond to gambling as a public health issue [15] through: harm minimisation, health promotion and the political determinants of gambling consumption. While foundational public health texts neglected to name gender as a social and economic determinant of gambling practices and harm (e.g. [16]), it has since been argued that gender is highly relevant to harm reduction in this framework [3, 17–19].

In gambling studies, gender is mainly explored by examining gender differences in gambling practices/preferences and motivations. These analyses tend to be carried out at the level of the individual, without reference to gender as a social construct shaping gambling practices and harm [3, 20, 21]. As a result, the dominant view of gender differences in gambling paints a particular picture of women in relation to public health risk: Women who gamble regularly may have a higher susceptibility to gambling problems, although they gamble less than men overall [22]. Suggested contributing factors include women’s preference for gambling on continuous forms (e.g., electronic gaming machines—EGMs) with minimal face-to-face contact, and women’s higher levels of anxiety and depression [21, 23, 24]. Women’s gambling is described as a risk factor for child neglect [25–27], and neglect of broader family responsibilities and roles [28–31]. Women have been found to be more likely than men to use gambling as an escape, to avoid and/or address boredom, loneliness, social isolation, and depression (e.g. [32–34]). However, this research is equivocal [35], often drawn from clinical samples [32, 33], and thus neglects to consider that people’s gambling experiences and practices are shaped by context, time and place [36–38]. There are significant gaps in our current understanding of how women are affected by gambling across the spectrum of low-risk to problem gambling, and as affected others [18, 19, 39, 40]. Existing research has tended to either ignore women, or analyse their practices and preferences without reference to gender dynamics [20, 21]. Gender informed analyses have rarely been conducted, and gambling studies have given insufficient attention to gender as an analytical category and/or theoretical construct [1, 18, 41]. Important gender differences may have been missed and/or unhelpful gender stereotypes reinforced [20, 21], constraining harm minimisation and treatment efforts.

A smaller body of research, drawing on sociological and gender theory, identifies that a complex array of individual, relational, contextual, cultural and normative factors relates women and men to gambling and gambling harm differently. For example, bingo halls have long been associated with women carving out leisure time and space apart from men and familial demands [42]. Certain gambling practices and environments are infused with masculine assumptions, practices, expressions and implied values, e.g. playing poker and spending time in betting shops [43, 44]. Participation in the UK National Lottery can be woven in to working-class women’s enactment of caring roles in the family, seen as part of prudent management of a household budget to give one’s family the best chances of success [45]. Certain gambling environments are produced and marketed as ‘safe’ for women, who are subjected to gender specific marginalisation and violence in public spaces [41, 46, 47].

Research informed by feminist social constructionism focuses on exploring ways to improve the lives of women and the requirements for such changes to occur [48]. This involves identifying and challenging assumptions about women’s essential ‘nature’, and promoting the recognition of practices and experiences as influenced by many social factors including areas of inequality [49]. Accounts of women’s behaviours and harm are treated not as straightforward representations of what women think, feel, do and need, but as indicative of gendered social and cultural discourses and processes shaping their practices and experiences. Processes of normalisation involve the interplay of socio-cultural, environmental, commercial and political determinants which “influence how different gambling activities and products are made available and accessible, encourage recent and regular use, and become an accepted part of life” for women, their families and communities [50]. In dominant psychological therapeutic frameworks of health and intervention, experiences that align with psychological discourse tend to be validated, and change relies on the uncovering of essential ‘truth’ and healthfulness within individual selves rather than social change [51]. Collective experience of societal ills can be constrained as a resource for harm reduction: “Therapists become the repositories of the stories we used to tell each other. But therapists can’t tell anyone else because of confidentiality rules” [52]. This narrowing and individualizing phenomenon has been critiqued in addictions harm reduction more broadly, for example, more holistic discourses of health and wellbeing allow women to resist dominant addiction treatment ideology positioning their struggles as an individual condition:

While acknowledging her need to address her temporary inability to stop using drugs, Susan refused to accept an identity based on powerlessness and composed of character defects… Susan saw the world in terms of power, privilege and difference; claiming powerlessness was ‘what women have been doing for years’ [53].

Addictions treatment and self-help services (as well as researchers, government departments, and other stakeholders), produce authoritative knowledge statements about ‘addictions’ and ‘addicts’. These constructions may or may not align with the lived experience of women or others, including those who may not identify as either male or female. In gambling studies, few analyses drawing on feminism and/or social constructionism have been conducted. This limited research has highlighted how some of the complexities involved in navigating ‘normative womanhood’ (e.g. balancing personal preferences/needs with gendered expectations) place constraints on gambling recovery [54], and on relationships with others affected by gambling addiction [55]. It has elaborated the pleasure function of gambling in the lives of some women, in conversation with social environmental and familial possibilities [56]. It has also pointed out how psychological treatment concepts such as ‘co-dependency’ can operate to pathologise and discipline women [57–59]. Mazzoleni and colleagues referred to the double edged nature of ‘co-dependency’ for women affected by gambling harm as “legitimising [their] problems, while stigmatizing them as inept and needy” [60].

Two influential qualitative studies of the nature of gambling harm in New Zealand conducted over the past decade (involving total *n* = 165 people who gambled, family and affected others, community leaders, gambling and community support service providers, policy makers and academics) neglected to explore the role of gender. The Gambling Harms study commenced in 2014, to systematically investigate gambling-related harm in New Zealand, and to assess the aggregate burden of harm caused by gambling to inform public health responses (see [5]). The Pacific Impacts project was conducted in 2011, to explore gambling harm among families and communities of Pacific Island ethnicity in New Zealand (see [61]). The absence of gender as an explicit focus of investigations of gambling harm in a public health framework, is indicative of the extent to which gender issues have been overlooked by gambling studies in recent years.

To address a lack of theoretically informed gender analyses identified in gambling studies of women (and men), this study revisited data collected in these two studies, with a feminist social constructionist lens. The aim of this study was to identify and explore gender-related issues, notions and practices, in relation to gambling harm for women. Key questions guiding this inquiry included: What gender or gender-related issues, notions or practices were discussed in relation to gambling harm? What are the implications of gender-related issues, notions or practices for women’s experiences of gambling harm? What are the implications of gender-related issues, notions or practices for women’s gambling harm reduction?

## Methodology and methods

### A feminist social constructionist perspective

To examine how gender-related issues, notions and practices may influence experiences of gambling harm this study drew on feminist social constructionist understandings of gender, gambling practices and harm. Gender categories were understood as constituted through sociocultural processes which shape men (‘masculinities’), women (‘femininities’) and non-binary genders [62]. This perspective draws particular attention to the mechanisms through which the social construction of gender impacts on the wellbeing of women who position themselves (or who are positioned) within and/or outside of normative boundaries [63]. As Holdsworth et al. [18, p. 209] stated in relation to gambling and harm, “gender is more than a source of personal and social identity; it is a key determinant in the social stratification system and for the distribution of resources within society”. Gambling is considered as a form of popular culture with links to the cultural dimensions of gender inequality and patriarchal power [56].

### Data selection

Two existing qualitative data sets related to gambling harm in New Zealand were identified as constituting a rich source of information regarding the gendered nature of gambling harm for women, as well as potential overlap/interactions with cultural identity (Gambling Harms Study and Pacific Impacts Study). The Gambling Harms study (see [5]) produced a large qualitative dataset, detailing New Zealander’s definitions, knowledge, experiences and ideas about gambling harm. Three focus group interviews were held with 26 participants comprising professionals involved in the provision of problem gambling treatment and allied support services (budget advice, social support), consumer representatives, regulators and academics. Eight focus group interviews and six individual interviews were held with a total of fifty-one individuals (25 female) comprising community members and treatment seeking individuals who identified that they had experienced harm from either their own, or someone else’s gambling, and with staff of gambling treatment services. This study involved participants from all the 4 main ethnic groups in New Zealand (i.e., those of Indigenous Māori, Pacific Island, Asian and European heritage). The Pacific Impacts dataset (see [61]) comprised twelve focus groups with ninety-two key Pacific stakeholders (61 female) including gambling treatment providers, gambling venue staff, general community gamblers and non-gamblers, current/ex-problem gamblers, significant others of problem gamblers and church leaders.

In both studies, recruitment of participants was purposive in relation to cultural identity/lens and relationship with gambling harm (e.g. whether a participant identified as a person who gambled, an affected other, or in a professional and/or community gambling harm reduction role). For example, in the Gambling Harms Study one focus group involving people who gamble was convened exclusively with those who identified as Māori (Indigenous peoples of Aotearoa New Zealand), another group of gambling support service providers involved specialists in the provision of culturally informed support services for Māori, Pacific and Asian peoples. In both studies, participants were guided to discuss definitions of ‘gambling’ and ‘gambling harm’, issues and impacts of gambling specific to individuals, families and communities, as well as any culture-specific relationships with gambling practices and harm.

### Data analysis

Step by step accounts of how to go about secondary analyses of qualitative data are rare. This approach drew on Bishop’s [64] reflexive account of reusing qualitative data. Data were coded using an iterative framework method [65], for sections of text where participants (men and women) were discussing gender (e.g. the nature of gambling harm for women or men) or gender-related issues, notions or practices (e.g. motherhood, fatherhood, women’s ‘escape gambling’). The aim here was to gather examples of how gender-related issues, notions and practices were intertwined with some accounts of gambling harm, where these existed in the data. One third of the transcripts were coded independently by two researchers. An internal data auditing process was performed [66]: after 10, 20 and then 30 transcripts had been coded, three members of the study team met to review a selection of transcripts and workshop the emerging coding framework.

Gender-related issues, notions and practices identified during the coding process were used as a springboard for thematic analysis. Themes were identified using the six phases identified by Braun and Clarke reflexively to bring a feminist social constructionist lens to bear on the data [67]. This involved discussion of the ways in which the social construction of gender and gambling may influence and shape women’s experiences of gambling harm and have implications for harm reduction. Language used to describe the relationships between gender, gambling and harm was not treated as conveying underlying ‘authentic’ or ‘real’ experience or cognitive states internal to participants (men and women). Rather, accounts were investigated for links with social and cultural conditions of possibility for experience to be articulated and given meaning [68]. As such, dissenting accounts were brought to the fore, even where few participants articulated them, as indicative of a range of socially produced possibilities and constraints operating on women’s gambling practises and harm.

## Results

Three broad themes were identified describing the social construction of gender and women’s gambling and harm. Women’s socially prescribed responsibility for domestic and emotional labour was particularly prominent, in addition to the impact of the role of motherhood on gambling and harm. These two themes referenced the social construction of men and men’s roles against which women were often defined and distinguished. Holistic discourses of health and wellbeing identified gambling and harm for women as linked to gendered poverty, patriarchal familial and social structures and processes of colonisation. Each theme is outlined below, followed by a discussion of the implications of this analysis for women’s gambling harm reduction/prevention activities.

### ‘It was special time for me’: women as family health and wellbeing managers

Primary responsibility for the domestic and emotional labour that keeps families functioning was placed on women, through repeated citation of traditional gendered roles of wife, mother, sister or daughter. Domestic labour included cooking and cleaning, organising and administrating a household, which often included multiple extended family members. Emotional labour involved being ‘a shoulder to cry on’, caring for others’ wellbeing, resolving disagreements between family members, and supporting family members dealing with trouble in all areas of their lives:

From a mother’s perspective I run everything in my family. I make sure I pay all my bills and make sure that everything’s done for the kids, everyone has lunch and stuff. (Female community gambler, Pacific Impacts Study)

…it's normally the wife that's the dominant person in the relationship for keeping the family together, she's the one that makes sure that everyone’s OK, there's food on the table, that all the bills are paid and the children are well looked after. (Pacific service provider, Harms Study)

Women were positioned as naturally best suited to caring work carried out in families, through innate emotional and interpersonal literacy:

In our family the women are always talking and working things out for everybody - I think we got that from our Aunties down the line… We sit amongst each other and we always talk this stuff out. As to my brothers, when they have an issue, it just sits so heavy on their chest or it comes out when they're drunk. (Female client of Māori gambling service, Harms Study)

I give all the time, I mean I'm everything for everybody, but nothing for myself and that’s, I just think women are naturally like that… (Female gambling support service user, Harms Study)

Responsibility for domestic and emotional labour shaped gambling practices, as women searched for space to be alone, to relax, apart from the requirements of others. Gambling opportunities in local communities were well-placed to fulfil this need, and therefore to become essential to everyday life:

Go to housie [bingo], nobody calls us Mum. Go to the pokies [EGMs], there's no one to say “Mum can I?” Or “Hon, where's the remote? Where's my jeans?” For me it purely became about that, the gambling. Definitely for someone to grab an addiction so fast- you're missing something in life… In my family it was like my dad had four wives. I was the second eldest child, but I'm the girl. (Female client of gambling support service, Harms Study)

Gambling on EGMs, in a separate room at local community pubs/bars, provided a space where women could be both physically and emotionally separate from familial demands for a period of time, in a way that could be scheduled around often severe constraints on their leisure time:

I'm held as the mother of the family, and I've got a big family. It's not just my family, it's my partner's family, and everybody relies on me. So I suppose when I got into gambling, I thought it was something for me. I thought it was special time for me. (Female client of gambling support service, Harms Study)

I don’t get to gamble like just when I want to. I have to put a certain amount of time, on the side of my week, which day it’s gonna be, where I can go, and if I know the kids are being looked after by my husband and the shopping’s already done. That’s my time. That’s my 2 hours or, or an hour and a half depending on how much time I’ve got. (Female general community gambler, Pacific Impacts Study)

Women’s social positioning as family health and wellbeing managers, coupled with a lack of alternative relaxing activities, produced EGM gambling as a strategy to process feelings of ambivalence, anger, and resentment towards families. Gambling could be regarded as a less harmful way of coping with such familial distress than drinking alcohol, especially for mothers or women with family members at home who depended on them.

It’s not as if my wife always goes to the [electronic gaming] machines… it’s only when there’s something she’s not happy about in my family or our relationship, she spends the money. It’s as if spending the money on that is her way of dealing with any anger towards the family. (Male significant other client of gambling support service, Pacific Impacts Study)

It’s about whānau [family] relationships. You might have had an argument… and you think “well stuff this, I'm off” - I might as well go and try the pokies [EGMs]… rather than go to the pub and drink, which messes you up for the rest of the day, I'll go and have a gamble. (Female client of Māori gambling service, Harms Study)

A gendered social meaning of community-based EGM gambling was constructed—one that recalls the historical positioning of certain prescription drugs and forms of alcohol as “Mother’s little helper” [69]. Women also described how a social responsibility to care for others produced a deep sense of personal responsibility for addressing gambling harm in their families. One participant described terminating a pregnancy in order to ensure she had the energy to support her gambling partner, who left her little capacity to consider taking care of a child. Harm caused to women by others’ gambling was described in visceral terms, as continuous feelings of worry and concern, stomach ulcers, sleepless nights, lack of sleep, migraine and feelings of exhaustion. Women described feelings of intense self-blame, shame and embarrassment when they were unable to address gambling harm through their caring roles:

I always feel like: Where did I go wrong, in my responsibility? I wasn't able to instil our values… I've failed because she's like a daughter to me and I'll be embarrassed for the rest of my life. I don't talk about this to anyone. (Female affected other, Harms Study)

In contrast, men’s roles were more often positioned as ‘advisors’ for loved ones affected by gambling problems, as opposed to ‘carers’—reinforcing a gendered logic/emotion dichotomy—and placing ultimate responsibility for improvement on the person with the gambling problem:

Males probably tend to deal with issues on a logical basis rather than emotionally… Females, more on a high emotion basis, whereas guys like me sit down and say, okay we need to do this, this, this and this and run through the steps logically. (Male affected other client of gambling support service, Harms Study)

Emotionally I would say no, my wife’s gambling didn't really affect me that much. Probably because I spent my time studying what's available and getting a better understanding of addiction… So that enabled me to provide solutions and get her to understand that you've got a problem and this may be the solution to help mitigate the problem. (Male community affected other, Harms Study).

While positioning women as responsible for addressing the psychological and emotional complexity of gambling harm in families, gender normativity seemed to operate to absolve some men of this responsibility, contributing to women’s sense of isolation and shame.

### The worst harm is to the kids’: women’ who gamble as ‘negligent mothers’

Within both studies, neglect of children was constructed as the most severe form of gambling harm possible. Harm to children was also constructed as a deeply emotional/psychological phenomenon: linked to being asked to keep family secrets, a lack of capacity to understand the ‘adult world’ of gambling and addiction, as well as witnessing arguments and violence in the home and the threatening behaviour of others (such as loan sharks). These experiences were seen to be ‘internalised’ by children, leading to self-blame, shame, insecure attachment styles and long-lasting mental health issues. While mothers and fathers were both implicated in children’s gambling harm, again the responsibility for causing and addressing the harm experienced by children was constructed differently for men and women through the intersection of social roles of fatherhood and motherhood. When gambling harm for children was described in relation to fathers, the most usual harms were not being able to provide for the family and not being able to spend enough leisure time with the children. For example: “My kids are running around, I probably should be playing with them rather than sitting in front of the computer” (Male general community gambler, Harms study).

In contrast, mothers were positioned as the primary caregivers for children in their day-to-day lives. Consequently, women’s gambling was much more often judged against the effects it had on children—e.g. “She hasn’t upset them or taken anything that the children own” (Male affected other client of gambling support service, Harms Study). Mothers were positioned as responsible for being with children at home. This included for example preparing food and picking the children up from school.

Mothers who gambled and neglected their mothering roles, were identified as causing the most severe emotional harm to their children. Parental quality was more easily questioned when it was the mother in the family who was gambling. Indeed, gambling was portrayed as deeply incompatible with being a loving mother, good wife and manager of household resources and family wellbeing:

Her husband couldn't believe that the woman he'd married turned out to be a gambler. He wanted to put ‘gambler’ on her gravestone. And the two boys, who idolised her, couldn't reconcile their mum who was down there [at the casino] hanging on to the drip… Well they felt quite betrayed. They thought they knew their mum really well, that she was a loving mother - but she just had to go up to that casino. (Community support worker, Harms Study)

Gambling was seen and experienced as particularly transgressive for mothers through societal, cultural and familial expectations that they prioritise their children’s needs. That women as mothers were judged more harshly in relation to harm experienced by children, had clear implications for women’s help and support-seeking.

For me, personally, going from a mum and doing all that life, and then becoming a gambler, right to the last $2, and transferring my children's money over and stuff like that, and then thinking, have they got money saved? … Then just feeling emotionally sick, to the point where I couldn't even cry because I knew it was unforgivable and all my fault what I was doing. (Female client of Māori gambling support service, Harms Study)

Women feared losing their children if gambling problems in the family, combined with their inability to cope on their own, became known—regardless of whether the gambling behaviour in question was their own or another’s. The social positioning of women as responsible for child wellbeing, placed women who experience gambling harm in their families, in a position where speaking about the harm was likened to a ‘confession’: “Yeah, confession is the hardest thing, yeah. As a mum, you lose your children” (Female general community affected other, Pacific Impacts Study).

### ‘You’ve got a hopeful not harmful mother there, okay’: women’s gambling and harm as socially produced

A minority of participants identified and challenged the construction of mothers who gamble as ‘negligent’, by drawing on holistic health and wellbeing discourses. These participants emphasised the structural and environmental factors that shape women’s gambling and harm:

You’ve got a hopeful not harmful mother there, okay. And the hopeful mother has $20… that mother is contemplating trying to feed five mouths, put food on the table… she will be practising gambling for a different kind of approach - to protect her kids. (General community participant, Pacific Impacts)

When poverty places the ‘hopeful mother’ in an already impossible situation ($20 to feed five mouths), gambling and the possibility of winning enough money to provide food for her children can become a form of care in some social and cultural contexts. EGM gambling can then be viewed as an available practice to increase the ability of some women to fulfil caring roles/expectations and maintain or accrue social and cultural capital. From a holistic health and wellbeing perspective, processes of colonisation producing whānau/family disconnection and disempowerment were identified as constraining indigenous Māori mothers’ wellbeing:

[Through] the historical trauma and the impact of colonisation, we’ve lost a lot of our cultural strengths, they’ve been systematically stripped by oppression. So we don’t have those systems in place - where we have the backups. Especially urbanised Māori, they don't have close family living nearby where they can say “Oh aunty can you watch the kids, mum’s going out for a while.” This day-to-day support for Māori women is not happening in an urban context. (Participant in government policy/academic focus group, Harms Study)

The multiple ways in which mothers may be constrained in their ability to live up to the social expectations surrounding ‘good mothers’, who always protect their children from gambling and harm, were identified as the primary issues to be explored and addressed in relation to harm reduction. Poverty and colonisation, patriarchal familial and social structures were identified as creating the conditions of possibility for some women to be threatened and intimidated into providing funds for gambling, and remaining in relationships with gambling men:

I feel sorry for the mothers, you know, cause in some families, the men they got the power, and they just demand, “Give me the money, I’ll do whatever I wanna do with it. (Female general community affected other, Pacific Impacts)

He would turn up demanding money because he didn’t have any petrol, couldn't get to work and it was quite menacing at the time. But then it didn’t fit into the ‘battered women syndrome’ as far as the women's refuge go, because he didn't actually ever hit me, but there was that intimidation that he needed money and he needed it now and who else was he going to get it from? (Female general community affected other, Harms Study)

Men’s coercive and controlling behaviours were identified as limiting some women’s personal autonomy, and ability to care for themselves, their children and other family members in relation to gambling harm, causing and exacerbating shame and isolation. Conversely, community EGM gambling environments (i.e. local bars/pubs) offered a safe, quiet, easily accessible activity, to spend time away from situations of coercion and abuse within the family and harassment in broader society:

But where it started was that I met up with this other tāne [man] and he was a heavy drinker, quite abusive verbally. Not so much but hitting me like my ex did - but I sort of started rebelling, getting away… I just one day went to the pub and this old Māori lady said to me – “try this” [EGM] - and I said I have no idea how to play it, but she showed me how to do it... I suppose that it started from there before it got out of hand (Female gambler, Māori community support service, Harms Study)

Some women also identified the impact of childhood abuse and historical intimate partner violence on the role that gambling played in their later lives to help them manage trauma. The relationship between gambling and violence against women was exacerbated by socioeconomic and health inequities, and processes of colonisation. The imposition of Western ways of living, and Western models of health and wellbeing, were described as weakening community-based safeguards and support systems. For women in general and Māori and Pacific families particularly, gambling, violence, other addictions and mental health issues could reflect situations of poverty and low social and cultural cohesion:

In the old days domestic violence wasn’t a thing in Māori culture. There's a saying that it takes a whole village to raise a child… with the Māori culture, if you stepped out of line, if one person stepped out of line, whether it would be hitting a child or a woman, that person would be dealt to. It's not just by one person, it's by the whole village. (Male affected other client, Māori gambling support service, Harms Study)

There’s an Island way that, no one else can solve a mother and a father’s domestic [violence] but from their mother and their father… doing counselling for Family Violence is the Europeans way of solving things. It does not fit into the Pacific way of solving stuff. It has to be a holistic approach. You’ve got to look at it from the spirit, body and mind. You’ve got to cover all those and you bring in the whole community. (Pacific gambling support service staff member, Pacific Impacts).

Participants across all ethnicities advocated for holistic and community development-oriented activities to support women to address gendered socioeconomic and health inequalities and gambling harm together. For example, one Pacific woman spoke about support groups run through Churches specifically for women to identify with and collectively advocate for women’s empowerment in Pacific communities. Other participants explicitly advocated for preventing gambling harm and other coexisting issues, as opposed to treating them after they have developed. These participants could link the notion of health promotion to creating healthier family environments free of poverty, racism and sexism for the benefit of future generations, and a specific need to prevent gambling harm through the empowerment of women and community connectedness. In doing so they referenced the Māori notion of kaitiakitanga—the process and practices of protecting and looking after the environment. As a concept, kaitiakitanga can align with a public health focus on shaping the environments in which health is produced [70].

## Discussion

Gambling venues are often positioned as ‘family friendly’, in ways that belie the potential for harm to occur [47]. For example, in New Zealand a large and popular Auckland venue has invited locals to “Join our family gathering” in ways that appeal particularly to women, e.g.: “bring your own meat and food over and our friendly chef will cook it for you” [71]. The results of this study highlight how the social construction of gender, power and privilege in families and communities can shape gambling practices and harm for women. Women’s social positioning as responsible for family wellbeing alongside a lack of safe, quality and easily accessible community-based recreation and support, has made EGM gambling a viable strategy for relaxation and processing feelings of resentment towards families. We3 found that the social construction of women’s responsibility for addressing harm in families, e.g., protecting children, can be so powerful that speaking about gambling harm was positioned as a ‘confession’, invoking notions of individual penance or punishment for one’s personal failing as a wife, mother or sister. Gender normativity could operate to relieve men (and broader society) of this harm reduction role, heightening women’s sense of isolation and shame. A minority construction of women’s gambling (and harm) as socially produced identified poverty, colonisation and patriarchal family and social structures as placing unreasonable expectations on women, compromising their wellbeing, weakening community connections and safeguards, and subjecting some women to coercion and violence. These social processes allowed EGM gambling venues in community settings to offer a sense of safety and security. This analysis supports Schüll’s [72] suggestion that gambling harm for women “is symptomatic of unresolved anxieties and tensions surrounding the place of care in our discursively individualist society”. It also suggests that women’s gambling practices and harm are shaped by poverty, patriarchy, and colonisation in the context of gendered responsibility to care.

Broader women’s health and leisure research identifies how ‘the family’ can be a problematic space for women regarding health and wellbeing. Women’s disproportionate responsibility for domestic and emotional labour produces gendered health inequities, e.g. as shown in recent nationally representative Australian population research involving six waves of data collected from Australian adults (*n* = 3828 men; 4062 women; 24–65 years) [73]. These researchers showed how women’s non-work time was so constrained, that it significantly lowered the point at which paid work hours affected women’s health (relative to men) because of time conflict, fatigue and stress. Boys and men can experience profound difficulties participating actively in families, and sharing the tasks of providing emotional intimacy or personal care that are integral to family life, wellbeing and processes of recovery [74]. Women continue to be positioned as primary carers in families, through representations of guilt, responsibility, work—family balance issues, and dominant forms of masculinity [75]. Challenging patriarchal family structures and practices, in ways that effectively support women’s autonomy and are culturally nuanced and appropriate, remains an ongoing challenge for public health promotion in New Zealand and worldwide [76].

**Fig. 1:**
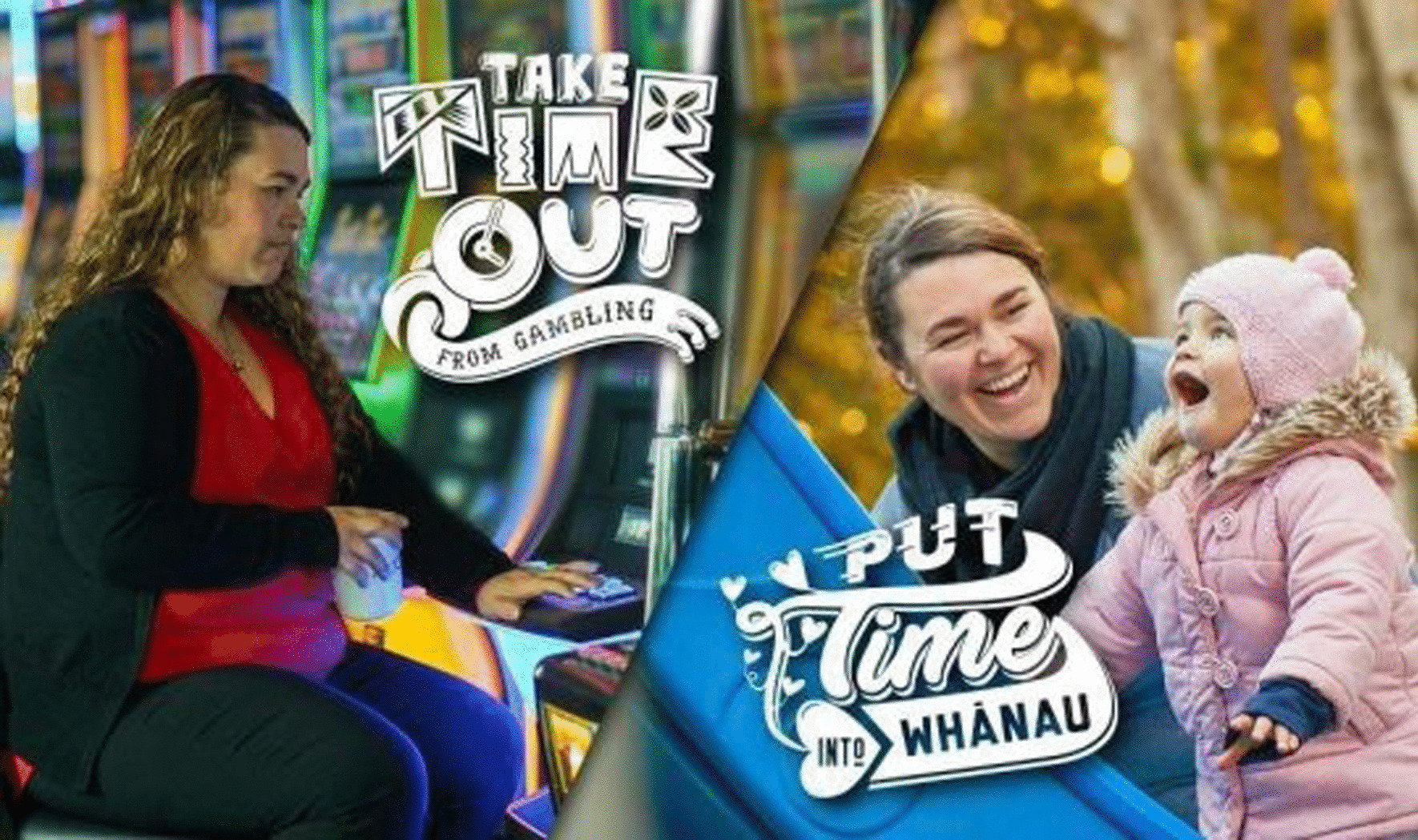
Health promotion imagery reproduced from HPA website [81]

Given that the social construction of gender, power and privilege in families and communities can shape gambling practices and harm for women, these factors must inform public health promotion and harm reduction practice. This forms part of the public health imperative to explore the role of community environments in shaping consumption behaviours and harm amongst different population subgroups [77]. Promoting gender equality is recognised as a key strategy for improving the health and wellbeing of women and their communities worldwide [78]. This includes efforts to increase women’s political, social and economic status and agency, ensuring participation and equal access to resources in society, so that women can determine the course of their own lives [79].Multiple international guidelines for gender-aware and gender-responsive health research and practice exist (e.g. [80]). The World Health Organisation (WHO) supports multiple layers of gender analysis in health research to support gender-responsive policy, accounting for personal and community-level impacts of gender, and investigation of the interactions between sex and gender and their dual impact on health. These tools and strategies do not appear to be routinely engaged in gambling harm prevention and reduction work. In the absence of explicit directive to examine inequalities, current public health practice is unlikely to be adequately responsive to gender-related issues [78, 80]. For example, the New Zealand Government Health Promotion Agency (HPA) responsible for minimising gambling harm, currently focuses on: “increasing the number of at-risk gamblers who check whether their gambling is okay, motivating at-risk gamblers to use appropriate self-help approaches and seek professional help when needed, and increasing the use of appropriate harm minimisation practices in gambling environments such as pubs and clubs with pokie machines” [81]. The strategies and practices of the HPA in recent years, have increasingly focussed on individuals experiencing problems (as encapsulated by the invocation of personal choice in the campaign slogan “Choice Not Chance”) [82]. Current national health promotion messaging and imagery targeting women encourages them to ‘put time into family/whānau not pokies [EGMs]’ (e.g. Fig. 1).

The notion of ‘choosing family not pokies’ reinforces powerful cultural and societal narratives which produce women as always-already responsible for familial wellbeing, and the strain this can place on some women’s mental and physical health [73, 83–85]. This messaging is concerning in the context of the findings of this study, where gendered coercion and violence within the family, as well as being overburdened with responsibility for family (and child) wellbeing, were found to be contextual factors for women’s problematic gambling and harm. The notion of ‘choosing family not pokies’ reinforces powerful cultural and societal narratives which produce women as always-already responsible for familial wellbeing, and the strain this can place on women’s mental and physical health [73, 83–85]. It also ignores the notion (fostered and promoted by the gambling industry) that gambling venues are ‘family friendly’ community spaces. Our research suggest that such health promotion strategies operating without cognisance of these gender dynamics have the potential to exacerbate or cause women harm. For example, women’s gambling harm advocate Brenda McQuillan has stated that when she was gambling on the pokies: "[Family] was all I thought about, I never stopped thinking about my family" [86].

This disconnect suggests that a return to earlier HPA (previously Health Sponsorship Council) conceptualisation of community ownership and influence over gambling harm is necessary, e.g. ‘problem gambling: our communities, our families, our problem’ (HSC 2009 cited in [82]). Action should be re-oriented to the conditions of possibility for gambling practices and harm: “getting society to understand the questions and issues around gambling harm” (HSC 2009 as cited in [82]). This includes supporting communities to conceptualise the social and political determinants of gambling harm, identify and action potential solutions [87, 88]. Health promotion has been given far less attention than harm minimisation in New Zealand and international research and practice [14, 82, 89]. Partnerships with New Zealand women’s health and gender equality organisations, in combination with theoretically informed and gender-sensitive research, may support and increase the quality of health promotion initiatives to reduce gambling harm for women [90]. Supporting gender equality aligns with key public health opportunities to address gambling harm: increasing community and broader societal accountability, and enhancing community engagement in decision making about health promoting/constraining environments [14, 82].

This study has presented evidence that women’s gambling harm is a social phenomenon, shaped by societal understandings and environments that affect women differently to men. Certain community gambling contexts (e.g. EGMs in local pubs and clubs) appear to play a particular social role for many women in the context of gender inequality, and therefore come with additional risk. EGM gambling can be conceptualised as a women’s health issue. These findings support the development of broader social interventions for gender equality, as well as critical work explicitly maintaining a dual focus on individual and social issues as responses to women’s gambling harm.

## Limitations

While data accessed for thematic analysis of experiences of gambling harm were not produced with gender analysis in mind, we hold that these studies did create space for many issues, notions and practices which have implications for gendered experiences of gambling harm to be discussed. It is possible that many more gender-related issues, notions and practices would be identified in data explicitly produced with this purpose and related questions. It is also acknowledged that the datasets accessed for analysis in this study are somewhat dated—collected between 2011 and 2014. The gambling environment has changed considerably in the last 9 years, for example online gambling is more available in New Zealand, and the uptake of online gambling has increased [91]. This may particularly affect women (and men) who can now gamble in the comfort of their own homes. This study has demonstrated the importance and value of understanding how gender-related issues notions and practices are related to gambling and harm. Future gender-aware research should keep pace with emerging and changing gambling forms. We understand that in the health equities field, performing secondary analysis is recognised as a useful way of reanalysing data that did not originally consider the concepts of sex and gender [92]. It provides the opportunity to explore previously unexamined dimensions of the research and ask additional questions not necessarily posed by the original researchers. We hold with Johnson and colleagues [92] that asking gender-related questions of any health related work is always relevant and useful, and can apply to any stage of the research process.

## Conclusions

This study has demonstrated the importance of integrating a broad awareness of how gender-related issues, notions and practices shape gambling and harm into all efforts to reduce gambling harm, particularly for women. This awareness is vital to avoid unwittingly contributing to stereotypical constructions of women and gender roles, which can constrain women’s health and wellbeing and access to resources and support. Key issues identified included: women’s socially prescribed responsibility for others’ wellbeing, disproportionate participation in caring work, and exposure to poverty, discrimination, violence, trauma and harassment. In the context of these issues, gambling venues in local communities appear to offer women respite, distraction, comfort, time-out and/or connection—while placing them at risk of experiencing problems and harm. Gender-blind public health activities run the risk of contributing to gambling harm for women. Theoretically informed gender analyses have the potential to make gender issues relevant to population groups visible, so that they can be addressed in public health promotion and harm reduction work. International guidelines for gender-aware and gender-responsive health research and practice (e.g. [80]) should be engaged as a foundation for strategic and effective harm reduction programmes, projects, research and policy, and as an essential part of developing and implementing interventions for gambling harm.

## Data Availability

The datasets accessed in this research are owned and administered by the New Zealand Ministry of Health. Availability is at the discretion of the New Zealand Ministry of Health.

## Abbreviations

EGM: Electronic gaming machine
HPA: Health Promotion Agency
WHO: World Health Organisation
